# Fluid Shear Stress Regulates Osteogenic Differentiation via AnnexinA6-Mediated Autophagy in MC3T3-E1 Cells

**DOI:** 10.3390/ijms232415702

**Published:** 2022-12-11

**Authors:** Tong Pei, Guanyue Su, Jie Yang, Wenbo Gao, Xinrui Yang, Yaojia Zhang, Jie Ren, Yang Shen, Xiaoheng Liu

**Affiliations:** Institute of Biomedical Engineering, West China School of Basic Medical Sciences & Forensic Medicine, Sichuan University, Chengdu 610041, China; ptdstudy@163.com (T.P.); sguanyue1124@163.com (G.S.); jiey_orthopedics@163.com (J.Y.); gaowb1018@163.com (W.G.); yangxinrui@stu.scu.edu.cn (X.Y.); yaojiazhang73@gmail.com (Y.Z.); rjstudy@163.com (J.R.); shenyang@scu.edu.cn (Y.S.)

**Keywords:** annexinA6, fluid shear stress, autophagy, osteogenic differentiation, mineralization

## Abstract

Fluid shear stress (FSS) facilitates bone remodeling by regulating osteogenic differentiation, and extracellular matrix maturation and mineralization. However, the underlying molecular mechanisms of how mechanical stimuli from FSS are converted into osteogenesis remain largely unexplored. Here, we exposed MC3T3-E1 cells to FSS with different intensities (1 h FSS with 0, 5, 10, and 20 dyn/cm^2^ intensities) and treatment durations (10 dyn/cm^2^ FSS with 0, 0.5, 1, 2 and 4 h treatment). The results demonstrate that the 1 h of 10 dyn/cm^2^ FSS treatment greatly upregulated the expression of osteogenic markers (Runx2, ALP, Col I), accompanied by AnxA6 activation. The genetic ablation of AnxA6 suppressed the autophagic process, demonstrating lowered autophagy markers (Beclin1, ATG5, ATG7, LC3) and decreased autophagosome formation, and strongly reduced osteogenic differentiation induced by FSS. Furthermore, the addition of autophagic activator rapamycin to AnxA6 knockdown cells stimulated autophagy process, and coincided with more expressions of osteogenic proteins ALP and Col I under both static and FSS conditions. In conclusion, the findings in this study reveal a hitherto unidentified relationship between FSS-induced osteogenic differentiation and autophagy, and point to AnxA6 as a key mediator of autophagy in response to FSS, which may provide a new target for the treatment of osteoporosis and other diseases.

## 1. Introduction

Bone remodeling is a dynamic process in which continuous bone resorption and bone formation adapt to mechanical stimuli in the environment of bones, and gives rise to a mature, intact, and stable bone structure [1]. Mechanical signals have profound impacts on bone mass regulation, bone homeostasis, and skeleton adaptation [2,3,4]. As an example, the loss of mechanical stimulation leads to the disuse osteoporosis [3,4]. Generally, mechanical forces are delivered to the bone tissues and sensed by mechanosensitive cells (osteocytes, osteoblasts, osteoclasts, and their progenitors), resulting in osteogenesis via cell proliferation, differentiation, and apoptosis [5,6,7].

In native bone tissues, bone cells reside within a complex microenvironment consisting of different mechanical stimuli, such as fluid shear stress (FSS), matrix stiffness, or mechanical loads, which lead to specific biological functions [1]. The daily movement process can facilitate the generation of fluid flow in the marrow cavity and on the endosteal surface by dynamic intramedullary pressurization and by applying FSS, ranging from 0.5 to 3 Pa, to bone cells [7,8]. Many in vitro studies also verified that osteoblasts couldsensitively respond to a wide range of different shear stress, of which 0.5–2 Pa is the most commonly used range [7,9]. The appropriate stimulation of FSS greatly contributes to the development and reconstruction of bone tissues through the activation of the gene expression of Runx2 and Osterix, and the secretion of collagen type I (Col I), osteocalcin (OC), and alkaline phosphatase (ALP) in bone mesenchymal stem cells (BMSCs) and osteoblasts [10,11]. To date, much attention has been paid to the effect of FSS on bone remodeling [12,13], but less is known about the underlying molecular mechanisms, such as how FSS integrates with other processes, including autophagy, which contributes greatly to bone remodeling to stimulate the differentiation of osteoblasts.

Annexin A (AnxA) is a family of calcium-dependent phospholipid-binding proteins that are widely distributed in vertebrates. Generally, annexins perform their biological functions via intracellular signal transduction or extracellular vesicle secretion, which is easily influenced by mechanical cues [14,15]. So far, 12 kinds of annexins (AnxA1-A11 and AnxA13) have been found in vertebrate cells [16], among which AnxA6, well-characterized matrix vesicle (MV) cargos in mineralization, are highly abundant in bone tissues [17]. AnxA6 involves the coordination of various physiological and pathological extracellular matrix (ECM) mineralization processes by regulating membrane and cytoskeleton organization, membrane trafficking, and signal transduction [18]. Growing evidence indicates that the abnormal expression of AnxA6 in osteoblasts and smooth muscle cells leads to some pathological processes such as the development of osteoporosis [19] and atherosclerosis [20,21]. The expression and distribution of annexins in cells are highly sensitive to changes in the mechanical microenvironment, especially fluid flow [14], whereas the role of AnxA6 in FSS-mediated osteogenic mineralization is unclear.

Our previous studies identified that autophagy can respond to FSS stimulation in cancer cell migration and invasion [22,23]. Autophagy is also regarded as an essential process in osteogenesis and is highly related with annexins [24,25]. AnxA1, AnxA5, and AnxA6 contribute to the fusion of autophagosomes with lysosomes, AnxA2 regulates phagophore formation, and AnxA7 regulates GTPase activity [18]. Additionally, AnxA6 contributes to the process of autophagy by regulating the formation of vesicular lipid membranes and promoting cell exocytosis, which occurs for instance, in the early stages of autophagosome biogenesis [26]. Given the role of autophagy in the proliferation and differentiation of osteoblasts, and the involvement of AnxA6 in the autophagic process [27], we explore whether FSS could promote osteoblastic differentiation via AnxA6-regulated autophagy.

In this study, we aim to elucidate the role of AnxA6-regulated autophagy in FSS-mediated osteogenic differentiation. When 1 h of 10 dyn/cm^2^ FSS was applied to MC3T3-E1 cells, we observed the upregulated expression of AnxA6 and osteogenic-differentiation-related proteins. The genetic knockdown of AnxA6 impaired FSS-induced osteoblast differentiation, which could be restored by inducing autophagy. Taken together, our study reveals that AnxA6 contributed to FSS-induced osteogenic differentiation by initiating autophagy.

## 2. Results

### 2.1. FSS Induces Osteogenic Differentiation

**Figure 1:**
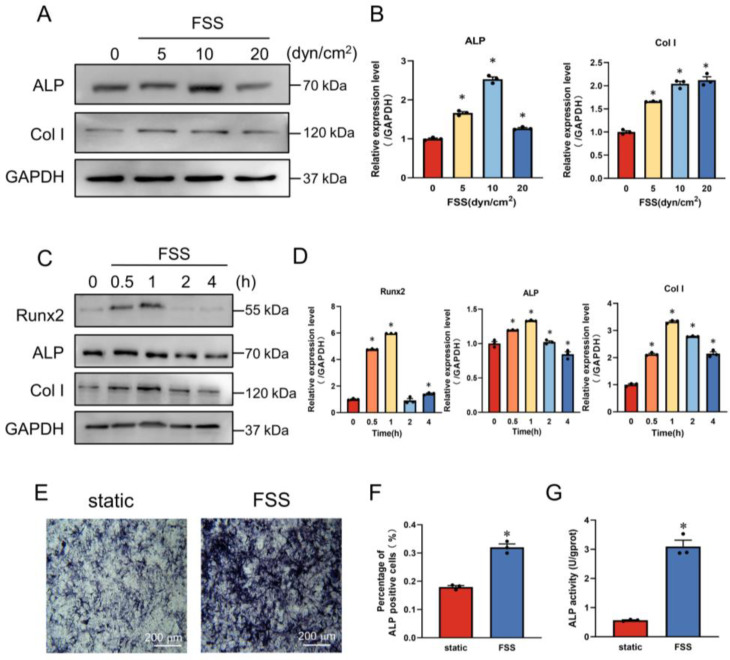
FSS induces osteoblast differentiation. (**A**,**B**) Western blot analysis and quantification of osteogenic protein expression in MC3T3-E1 cells when exposed to 0 (static control), 5, 10, pr 20 dyn/cm^2^ FSS for 1 h. GAPDH served as an internal control (*n* = 3). (**C**,**D**) Western blot analysis and quantification of osteogenic protein expression in MC3T3-E1 cells when exposed to 10 dyn/cm^2^ FSS for 0 (static control), 0.5, 1, 2, or 4 h. GAPDH served as an internal control (*n* = 3). (**E**) ALP staining after 5 additional days of osteogenic induction when exposed to 10 dyn/cm^2^ FSS for 1 h. (**F**) Statistical bar graph showing the percentage of ALP-positive cells in (**E**) (*n* = 3). (**G**) Quantification of ALP activity in MC3T3-E1 cells with or without 1 h of 10 dyn/cm^2^ FSS stimulation (*n* = 3). All data are presented as mean ± SEM. * *p* < 0.05 versus static control group.

To investigate the effect of FSS on bone formation, we first explored how FSS influences osteogenic differentiation of mechanosensitive cells MC3T3-E1. FSS with different intensities of 0 (static control), 5, 10, and 20 dyn/cm^2^, was applied to MC3T3-E1 cells for 1 h. The Western blot results show that FSS significantly promoted the expression of osteogenic markers, Col I and ALP in comparison with the control group (Figure 1A,B). Col I expression was upregulated as FSS intensity increased, with the highest expression observed on the group of 20 dyn/cm^2^, while the expression of ALP increased to its peak under the condition of 10 dyn/cm^2^. In order to optimize the effect of FSS on osteogenic differentiation, different loading times of 0 (static control), 0.5, 1, 2, or 4 h under an FSS intensity of 10 dyn/cm^2^ were applied to MC3T3-E1 cells. We observed that the 10 dyn/cm^2^ intensity of FSS provoked the expression of osteogenic proteins Runx2, ALP, and Col I, which increased to the highest levels after 1 h FSS treatment (Figure 1C,D). In addition, ALP staining and ALP activity in MC3T3-E1 cells were performed and monitored, respectively, to further explore the effect of FSS on osteogenic differentiation. As shown in Figure 1E,F, compared with the static group, ALP-positive cells increased by 1.8-fold when exposed to 10 dyn/cm^2^ FSS for 1 h. Consistently, ALP activity also showed a significant enhancement up to 3.6-fold (Figure 1G). These results suggest that the application of FSS with 10 dyn/cm^2^ intensity for 1 h is optimal for promoting the osteogenic differentiation of MC3T3-E1 cells.

### 2.2. FSS Promotes the Expression of AnxA6 in MC3T3-E1 Cells

**Figure 2:**
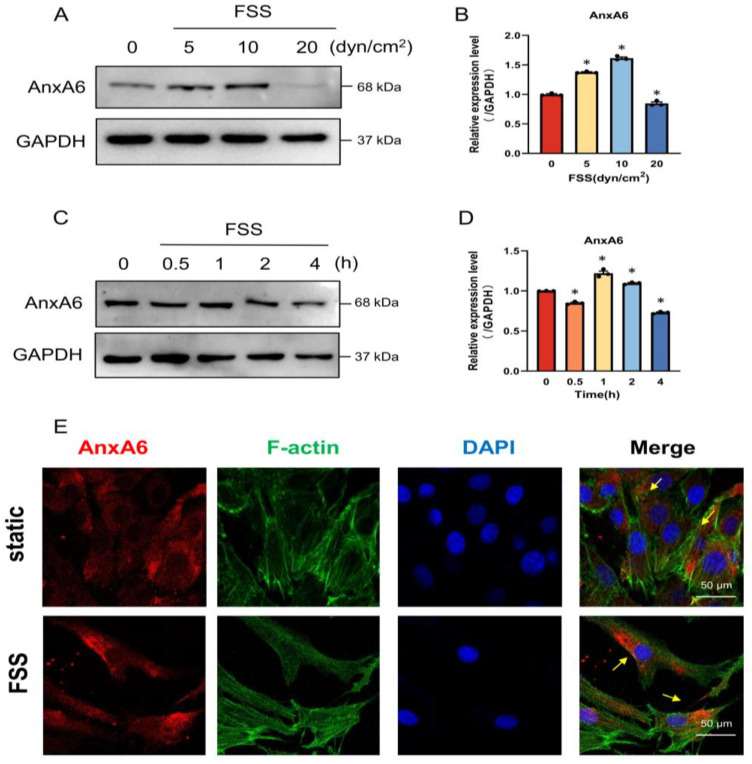
FSS promotes the expression and translocation of AnxA6 in MC3T3-E1 cells. (**A**,**B**) Western blot analysis and quantification of AnxA6 expression in MC3T3-E1 cells when exposed to 0 (static control), 5, 10, or 20 dyn/cm^2^ FSS for 1 h. GAPDH served as an internal control (*n* = 3). (**C**,**D**) Western blot analysis and quantification of AnxA6 expression in MC3T3-E1 cells when exposed to 10 dyn/cm^2^ FSS for 0 (static control), 0.5, 1, 2, or 4 h. GAPDH served as an internal control (*n* = 3). (**E**) Representative immunofluorescence images showing the expression and distribution of AnxA6 (labelled by red fluorescence, indicated by yellow arrows) inside the cell and F-actin organization (labelled by green fluorescence) when exposed to 10 dyn/cm^2^ FSS for 1 h. Nuclei were stained with DAPI (blue), scale bar = 50 μm. All data are presented as mean ± SEM. * *p* < 0.05 versus static control group.

AnxA6, as an important member of the annexin protein family that are abundant in bone tissues, plays a key role in ECM mineralization [28]. Previous studies showed a strong correlation between annexins and mechanical factors [14,15]. However, they failed to elucidate whether AnxA6 can respond to FSS during the process of osteogenic differentiation. In order to investigate the response of AnxA6 to FSS, 0, 5, 10, and 20 dyn/cm^2^ FSS were separately loaded on MC3T3-E1 cells for 1 h. As determined via Western blot, AnxA6 displayed a significant elevation under 5 and 10 dyn/cm^2^ FSS, but it decreased markedly when exposed to 20 dyn/cm^2^ FSS (Figure 2A,B). To further explore the effect of FSS-loading duration on AnxA6 expression, different loading times of 0, 0.5, 1, 2 and 4 h were applied to MC3T3-E1 cells under an intensity of 10 dyn/cm^2^ FSS. Compared to the static group, AnxA6 increased to the highest level after 1 h of FSS treatment, which is consistent with the previous results of the highest expression of osteogenic proteins, whereas AnxA6 expression decreased with 0.5 and 4 h of treatment (Figure 2C,D). Similar to the results of Western blot analysis, elevated immunofluorescence intensity of AnxA6 in MC3T3-E1 cells was visualized after treating with 1 h of 10 dyn/cm^2^ FSS (Figure 2E). AnxA6 accumulated to one side of the cytoplasm and showed a preference to the plasma membrane when exposed to FSS, which was different from the homogeneous distribution of AnxA6 in the cytoplasm observed in the control group (Figure 2E). These results demonstrate that 1 h of 10 dyn/cm^2^ FSS to cells can promote the expression of AnxA6, which is consistent with the expression of Runx2, ALP, and Col I shown in Figure 1, indicating a potential correlation between AnxA6 expression and osteogenic differentiation.

### 2.3. AnxA6 Involves in FSS-Induced Osteogenic Differentiation

**Figure 3:**
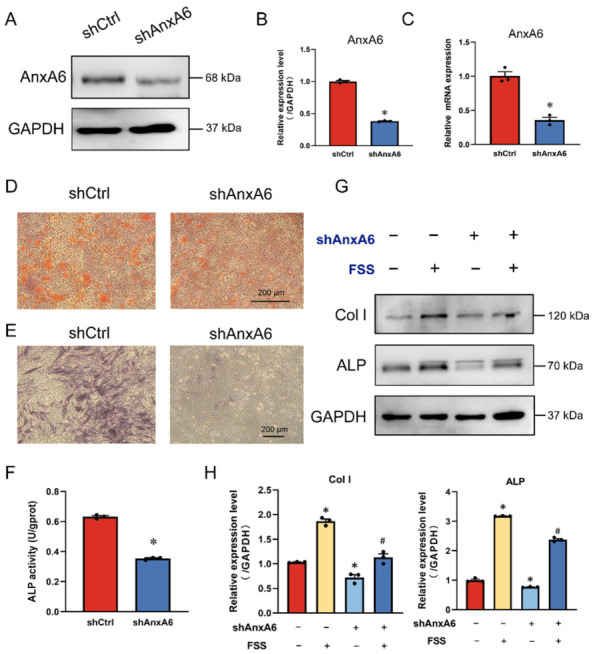
AnxA6 is involved in FSS-induced osteogenic differentiation. (**A**,**B**) Western blot analysis and quantification of AnxA6 expression in MC3T3-E1 cells transfected with negative shRNA (shCtrl) or AnxA6 shRNA (shAnxA6). GAPDH served as an internal control (*n* = 3). (**C**) The gene expression of AnxA6 was detected with qRT-PCR and quantified with GAPDH normalization (*n* = 3). (**D**) Representative images show alizarin red staining after 14 days of osteogenic induction. Calcified nodules were shown as red staining. (**E**) Representative images of ALP staining after 5 days of osteogenic induction. ALP-positive cells shown as blue staining. (**F**) Quantification of ALP activity in shCtrl and shAnxA6 MC3T3-E1 cells with 1 h of 10 dyn/cm^2^ FSS loading (*n* = 3). (**G**,**H**) Western blot analysis and quantification of osteogenic protein expression in shCtrl and shAnxA6 MC3T3-E1 cells with or without 1 h of 10 dyn/cm^2^ FSS loading (*n* = 3). GAPDH served as an internal control (*n* = 3). All data are presented as mean ± SEM. * *p* < 0.05 versus static shCtrl group; # *p* < 0.05 versus FSS group.

A stable AnxA6 knockdown MC3T3-E1 cell line was constructed to study the effect of AnxA6 on osteoblastic differentiation. Compared with the shCtrl group, the AnxA6 protein and gene were greatly downregulated in the shAnxA6 group indicating the successful knockdown of AnxA6 (Figure 3A–C). In addition, the deficiency of AnxA6 led to less mineral deposition (Figure 3D), fewer ALP-positive cells (Figure 3E),and lower ALP activity (Figure 3F) in MC3T3-E1 cells. The suppression of osteogenic differentiation from the absence of AnxA6 was further confirmed by the decreased expression of osteogenesis-associated proteins ALP and Col I (Figure 3G,H). To further explore the role of AnxA6 in FSS-regulated osteogenesis, 1 h of 10 dyn/cm^2^ FSS was applied to shCtrl and shAnxA6 MC3T3-E1 cells. As shown in Figure 3G,H, the expressions of ALP and Col I in shAnxA6 cells recovered to high levels when exposed to 1 h of 10 dyn/cm^2^ FSS treatment, but they still could not exceed those in shCtrl cells under the same FSS loading.

### 2.4. AnxA6 Knockdown Impairs Autophagy under FSS Condition

**Figure 4:**
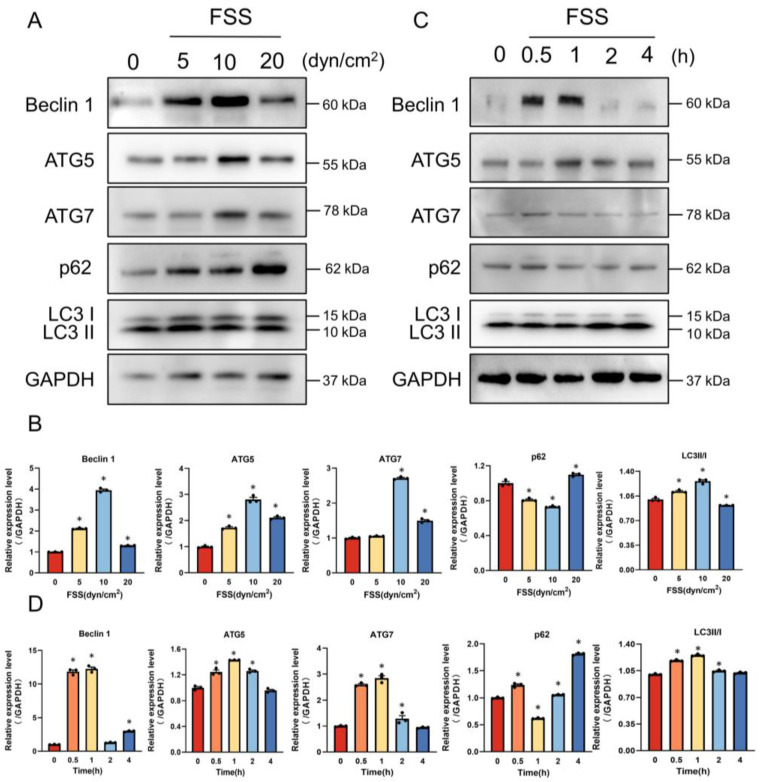
FSS induces the occurrence of autophagy in MC3T3-E1 cells. (**A**,**B**) Western blot analysis and quantification of autophagic protein expression in MC3T3-E1 cells when exposed to 0 (static control), 5, 10, or 20 dyn/cm^2^ FSS for 1 h. GAPDH served as an internal control (*n* = 3). (**C**,**D**) Western blot analysis and quantification of autophagic proteins expression in MC3T3-E1 cells when exposed to 10 dyn/cm^2^ FSS for 0 (static control), 0.5, 1, 2, or 4 h. GAPDH served as an internal control (*n* = 3). All data are presented as mean ± SEM. * *p* < 0.05 versus static control group.

Autophagy, identified as a necessary part of bone remodeling, can regulate osteoblast differentiation and mineralization [24]. Previous studies have shown that AnxA6 is involved in the process of autophagy, especially in the early stages of autophagosome biogenesis [26]. Primed by these findings, we explored whether FSS-induced AnxA6 could influence autophagy. Compared to the static group, cells exposed to 5, 10 and 20 dyn/cm^2^ FSS displayed higher expressions of autophagic markers Beclin1, ATG5 and ATG7, and were accompanied with more LC3B-I_to_LC3B-II transitions, indicating the accelerated occurrence of autophagy (Figure 4A,B). We further studied the influence of FSS loading time on autophagy by regulating the FSS duration. Compared with the static control, the expressions of Beclin1, ATG5, and ATG7 increased in MC3T3-E1 treated with 0.5 and 1 h of 10 dyn/cm^2^ FSS, while p62, which can be absorbed and destroyed in autolysosomes [29], decreased greatly after 1 h of FSS treatment (Figure 4C,D). These results strongly suggest that the application of 1 h FSS with 10 dyn/cm^2^ intensity to cells leads to a significant occurrence of autophagy. 

**Figure 5:**
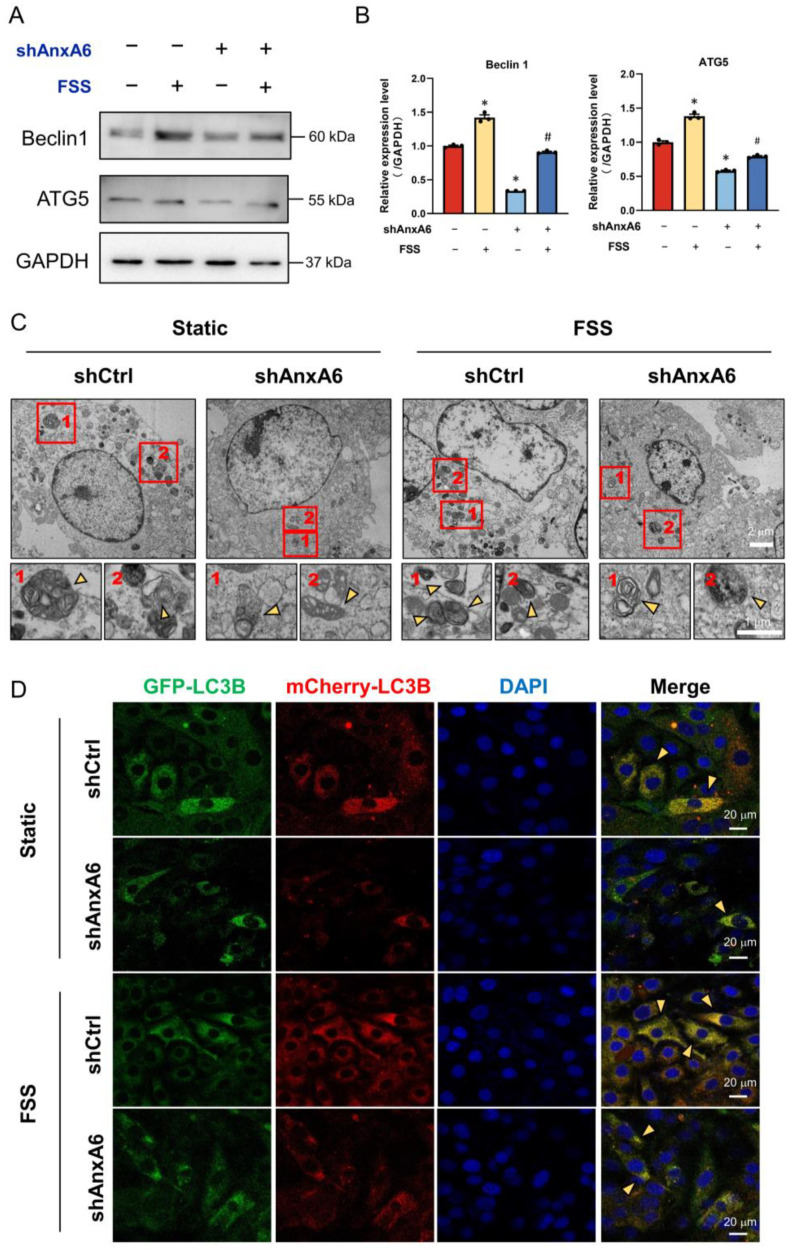
Knockdown of AnxA6 impairs FSS-induced autophagy. (**A**,**B**) Western blot analysis and quantification of autophagic protein expression in shCtrl and shAnxA6 MC3T3-E1 cells with or without 1 h of 10 dyn/cm^2^ FSS loading (*n* = 3). GAPDH served as an internal control (*n* = 3). (**C**) Typical TEM images of autophagosomes in shCtrl and shAnxA6 MC3T3-E1 cells with or without 1 h of 10 dyn/cm^2^ FSS loading. Autophagosomes are indicated by yellow arrows in the zoomed images. (**D**) Confocal images showing the distribution of LC3B during the process of autophagy in shCtrl and shAnxA6 MC3T3-E1 cells transfected with AdPlus-mCherry-GFP-LC3B adenovirus with or without 1 h of 10 dyn/cm^2^ FSS loading. Yellow mCherry-GFP-LC3B spots indicated by yellow arrows represent the overlap of mCherry- and GFP-LC3B showing the formation of autophagosomes. All data are presented as mean ± SEM. * *p* < 0.05 versus static shCtrl group; # *p* < 0.05 versus FSS group.

In order to reveal the role of AnxA6 in FSS-induced autophagy, we applied 1 h of 10 dyn/cm^2^ FSS to shCtrl and shAnxA6 MC3T3-E1 cells. We found that Beclin1 and ATG5 were significantly increased by FSS in the shCtrl group, and greatly suppressed in AnxA6 knockdown cells, regardless of FSS loadings (Figure 5A,B). Transmission electron microscope (TEM) is an effective tool to observe the morphology of autophagosomes and identify the occurrence of autophagy. The results of TEM in Figure 5C demonstrate similar trends with those of the Western blot analysis above. More autophagosomes were formed via FSS induction in shCtrl cells, whereas AnxA6 knockdown cells showed a decreased number of autophagosomes. In addition, an AdPlus-mCherry-GFP-LC3B infection assay was applied to track autophagosomes in MC3T3-E1 cells. The results in Figure 5D show more LC3B punctate dots in the cytoplasm when exposed to FSS, indicating the occurrence of autophagic flux, although the knockdown AnxA6 group impeded the process. All these results suggest that FSS can induce the occurrence of autophagy via AnxA6 induction.

### 2.5. FSS Promotes Osteogenic Differentiation by Activating AnxA6-Mediated Autophagy

**Figure 6:**
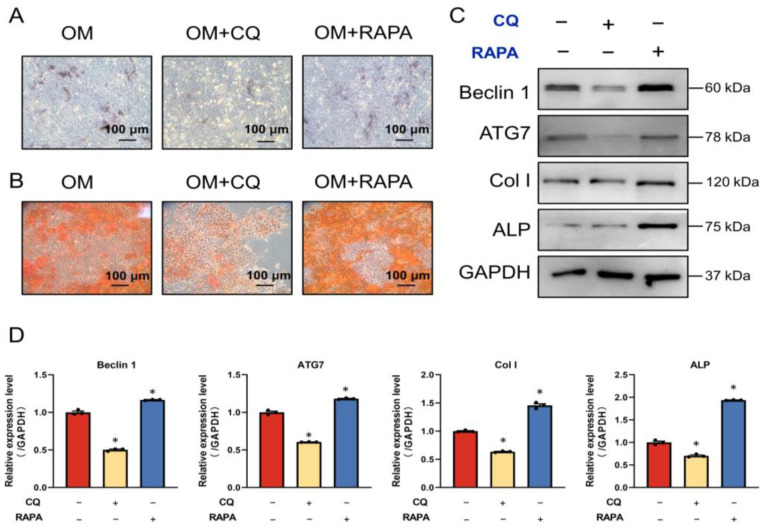

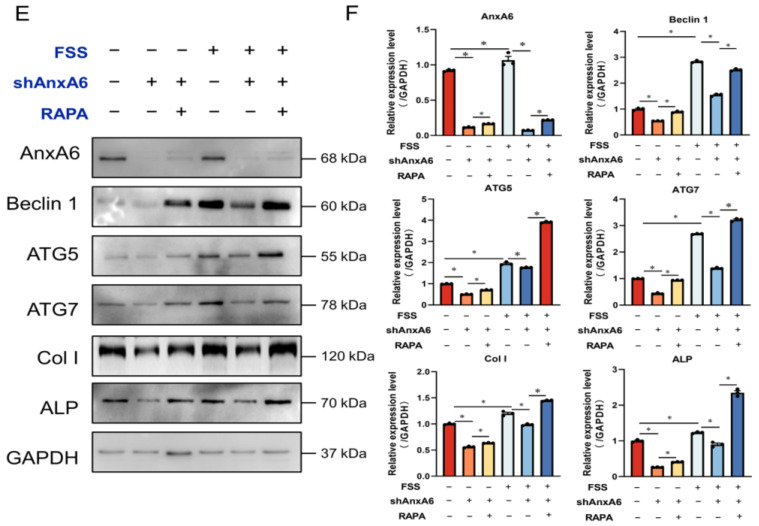
FSS promotes osteogenic differentiation by activating AnxA6-mediated autophagy. (**A**) Representative images showing the ALP staining of MC3T3-E1 cells after culturing in osteogenic medium (OM) with or without chloroquine (CQ, 10 μM) and rapamycin (RAPA, 100 nM) for 7 days. ALP-positive cells are shown as blue staining. (**B**) Representative images of the alizarin red S staining of MC3T3-E1 cells after culturing in an osteogenic medium with or without chloroquine (CQ, 10 μM) and rapamycin (RAPA, 100 nM) for 14 days. Calcified nodules shown as red staining. (**C**,**D**) Western blot analysis and quantification of autophagic and osteogenic protein expression in MC3T3-E1 cells after culturing in OM with or without chloroquine (CQ, 10 μM) and rapamycin (RAPA, 100 nM) for 7 days. (**E**,**F**) Western blot analysis and quantification of autophagic and osteogenic protein expression in shCtrl and shAnxA6 MC3T3-E1 cells with or without the pretreatment of RAPA (200 nM) under static or 1 h of 10dyn/cm^2^ FSS loading. * *p* < 0.05.

To further dissect the role of autophagy in osteogenic differentiation and matrix mineralization, we modulated the status of autophagy using an autophagic inhibitor (chloroquine) and an autophagic activator (rapamycin) during the in vitro mineralization process. As shown in Figure 6A,B, after culturing MC3T3-E1 cells with an osteogenic medium for 7 or 14 days, the inhibition of autophagy by chloroquine greatly reduced the number of ALP-positive staining cells, and inhibited the formation of mineralized nodules, while autophagic activation via rapamycin showed the exact opposite effect, promoting the mineralization process. Moreover, chloroquine addition decreased the expression of Beclin1 and ATG7, and the expression of ALP and Col I in MC3T3-E1 cells. On the other hand, rapamycin upregulated autophagy- and osteoblastic-differentiation-associated protein expression (Figure 6C,D). These results indicate that alterations in osteoblast differentiation and mineralization were evoked by autophagic regulation.

We next investigated whether FSS-induced AnxA6 enhanced osteoblast differentiation via activating autophagy. Since knocking AnxA6 down resulted in the suppression of autophagy and osteogenic differentiation under both static and FSS conditions, rapamycin was used to restore the autophagic flux suppressed by the AnxA6 knockdown and subsequently detect the expression of osteogenic markers in order to identify the effect of autophagy in this process. As shown in Figure 6E,F, the precondition of rapamycin recovered the expression of ALP and Col I, which had been inhibited in the shAnxA6 group, especially under FSS loading conditions. Moreover, the expression of ALP and Col I showed a positive correlation with the expression of autophagy markers, including Beclin1, ATG5, and ATG7. Overall, FSS contributes to the differentiation and mineralization of osteoblasts through AnxA6-mediated autophagy activation.

## 3. Discussion

Bones undertake a lifelong mechanical-loading associated remodeling process by balancing bone-forming osteoblasts and bone-absorbing osteoclasts. As one of the key components of the bone multicellular units, osteoblasts are specifically responsible for the mineralization of the bone matrix by responding to mechanical changes from body weight, movement and gravity [2]; for example, appropriate exercises contribute to enhancing bone density and preventing bone loss [30]. Sun et al. revealed that the application of FSS to osteoblasts in vitro increased the expression of osteogenic-differentiation-related proteins, and that mechanical loading from exercise could promote osteogenesis in vivo [4,31]. Microgravity and mechanical unloading strongly influence bone structures, leading to the dysfunction of osteoblasts and decreased bone mineral density. In this study, we found a clear link between the properties of FSS (density and duration) and osteogenic differentiation; 1 h of 10 dyn/cm^2^ FSS loading optimal for the differentiation and mineralization of osteoblasts (Figure 1). However, FSS-induced osteogenic differentiation is not always of FSS magnitude or duration-dependent. Higher stresses or a long duration may lead to damaging or negative responses, which was also reported in previous studies [32]. Accordingly, the proper magnitude and duration of FSS are vital for osteogenesis. Since osteoblasts sensitively respond to a certain range of FSS and contribute to bone formation, the underlying molecular mechanisms that greatly impact on the prevention and treatment of bone diseases, such as osteoporosis and fractures, need to be explored.

An elevated AnxA6 protein, which can respond to mechanical loading and influence cell proliferation [33], differentiation [28], migration [34], and other activities, was observed in our study under FSS loading (Figure 2). This finding is consistent with those of previous studies regarding the role of the AnxA protein family in mechanoresponses that demonstrated that the disturbance flow condition facilitated the interactions between AnxA2 and integrin α5 to activate integrin in endothelial cells [14], and that AnxA5 mediated mechanotransduction by detecting the calcium response of osteoblasts to oscillating fluid flow [15]. In our study, we found that FSS with different intensities and durations led to different responses of AnxA6. In detail, AnxA6 increased under 5 and 10 dyn/cm^2^ FSS loading, while it decreased with 20 dyn/cm^2^, suggesting the existence of an FSS threshold for AnxA6 induction. In general, AnxA is homogeneously distributed in the cytoplasm while responding to certain stimuli, such as glucocorticoids [35,36], inflammation [36], a high concentration of calcium [37], or FSS [14]. Annexins translocate from the cytoplasm to the plasma membrane to participate in signal transduction and perform corresponding functions. Our results in Figure 2E similarly reveal a translocation trend of AnxA6 from the cytoplasm to the plasma membrane under the exposure of 1 h of 10 dyn/cm^2^ FSS.

AnxA6, as an essential component in extracellular vesicles, is overexpressed in the zones of hypertrophic and terminally differentiated growth plate chondrocytes [38]. It plays a vital role in extracellular mineralization and is highly related to the development of osteoporosis. AnxA6 contributes to the accumulation of Ca^2+^ and stabilizes Ca^2+^-binding to phosphatidylserine (PS), leading to a favorable environment for apatite formation [39]. Given the effect of FSS on AnxA6 expression and osteogenic differentiation, we demonstrated here that the genetic knockdown of AnxA6 significantly inhibited osteoblast differentiation induced by FSS loading (Figure 3). However, the expressions of osteogenic differentiation markers were still higher than those of the shAnxA6 group. The possible reasons that may be attributed to that, besides AnxA6, are various other mechanoreceptors, including ion channels, integrins, connexins, G-protein coupled receptors, primary cilia, and cytoskeletons also existing in osteoblasts [1,40,41,42] and participating in mechanical-stimulus-regulated osteogenesis. Accordingly, AnxA6 is involoved in FSS-induced osteogenic differentiation and may serve as a potential mechanosensitive protein that can directly or synergistically respond to mechanical stimulation.

As reported previously, annexins are involved in multiple biological processes, including autophagy [18], epithelial-mesenchymal transition (EMT) [43], and extracellular matrix formation [44]. As an evolutionary biological mechanism, autophagy is closely associated with the metabolism, survival, and differentiation of osteoblasts [45,46]. It can promote the development of bones by stopping the calcification of endplate chondrocytes [47]. According to Liu et al., the inhibition of autophagy impairs osteoblast development and results in osteopenia in mice [48]. Conversely, activating osteocyte autophagy by rapamycin could reduce the severity of age-related bone changes in the trabecular bones of old male rats [49]. Zhang et al. found that FSS-induced autophagy in bone cells could regulate cell survival via the ATP metabolism [50]. Our previous studies also revealed that FSS could promote cell migration and invasion by activating autophagy [23,29,51,52]. In this study, we showed that FSS could stimulate autophagy by enhancing the expression of autophagic markers and the formation of autophagosomes, which was in full agreement with previous studies (Figure 4 and Figure 5). However, it is unknown how AnxA6 participates in FSS-induced autophagy during osteogenic differentiation, even though the contribution of AnxA6 to autophagy during cancer progression was confirmed [53,54]. According to our results, the genetic knockdown of AnxA6 could suppress the expression of autophagic markers even under FSS loading conditions (Figure 5). 

Given the fact that autophagy plays a central role in the coordination of bone development, we further proved the contribution of autophagy to osteoblast mineralization by using an autophagic inhibitor and activator (Figure 6A–D). Autophagic activation serves as an essential part of the cyclic mechanical-stretching-promoted osteoblast differentiation of BMSCs [55]. We demonstrated that the osteogenic differentiation inhibited by AnxA6 knockdown recovered greatly when pretreating with autophagic activator RAPA (Figure 6E), which is beneficial to ECM mineralization and bone formation. 

**Figure 7:**
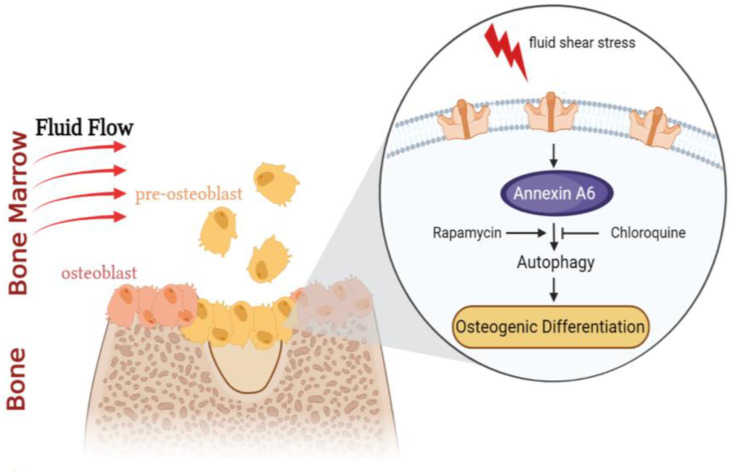
FSS promotes osteoblast differentiation via AnnexinA6-mediated autophagy. Pre-osteoblast MC3T3-E1 cells are sensitive to external mechanical stimulation. Once FSS is exerted on MC3T3-E1 cells, AnxA6 can respond to FSS directly or synergistically with other mechanosensors, accompanied by its translocation from the cytoplasm to the cytomembrane. Subsequently, the elevated expressions of AnxA6 affect the downstream autophagic flux, and further regulate osteogenic differentiation.

In conclusion, our findings indicate that AnxA6-regulated autophagy plays an important role in FSS-induced osteogenic differentiation, as shown schematically in Figure 7. We found that 1 h of 10 dyn/cm^2^ FSS providedan adequate amount of AnxA6 to initiate autophagy and increased ALP and Col I expression, leading to osteogenic differentiation. The knockdown of AnxA6 strongly inhibited autophagy and subsequently decreased osteogenic differentiation, while the restoration of the autophagic flux by an autophagic activator recovered the effect of FSS on osteogenic differentiation. All these results provide novel insights into the mechanical mechanisms underlying FSS-induced osteogenesis. However, one limitation of our study is that it is not clear whether AnxA6 can directly respond to FSS or be indirectly modulated by other mechanosensors. In addition, though the effect of autophagy in AnxA6-mediated osteogenic differentiation was identified in this study, the underlying molecular mechanisms are still obscure. Future studies may help in providing novel strategies for the prevention and treatment of osteoporosis.

## 4. Materials and Methods

### 4.1. Cell Culture

Murine osteoblastic MC3T3-E1 cells were purchased from Guangzhou Jennio Biotech Co., Ltd.(Guangzhou, China) and cultured with Dulbecco’s modified eagle medium (DMEM, High Glucose, Gibco, Grand Island, NY, USA), supplemented with 10% fetal bovine serum (FBS, Cell-Box, HongKong, China) and 100 U/mL penicillin-streptomycin (Hyclone, Logan, UT, USA) in an incubator with 5% CO_2_ at 37 °C. For osteogenic differentiation, 10 mM β-glycerolphosphate disodium salt hydrate (Sigma-Aldrich, St. Louis, MO, USA), 10 nM dexamethasone (Sigma-Aldrich, St. Louis, MO, USA), and 50 μg/mL L-ascorbic acid (Sigma-Aldrich, St. Louis, MO, USA) were added to complete the DMEM medium to the prepare osteogenic medium. 

### 4.2. Reagents and Antibodies

For the Western blot and immunofluorescence assays, the following were the primary antibodies: rabbit polyclonal anti-AnxA6; rabbit monoclonal anti-ATG5; mouse monoclonal anti-ATG7; rabbit polyclonal anti-Beclin1; rabbit polyclonal anti-p62; rabbit monoclonal anti-LC3B; rabbit polyclonal anti-ALP; rabbit polyclonal anti-Col I; rabbit monoclonal anti-Runx2; rabbit monoclonal anti-GAPDH; FITC Phalloidin(CA1620, Solarbio, Beijing, China). In addition, the secondary antibodies were as follows: goat anti-mouse (#L3032, SAB, College Park, MD, USA); goat anti-rabbit (#L3012, SAB, College Park, MD, USA); goat anti-rabbit, TRITC (ZF-0316, ZSGB-BIO, Beijing, China). The detailed information of primary antibodies is listed in Table 1.

For autophagic regulation, rapamycin (RAPA, 100 nM or 200 nM; Sigma-Aldrich, St. Louis, MO, USA) induced autophagy by inhibiting the activity of mTOR. Chloroquine diphosphate (CQ, 10 μM; Sigma-Aldrich, St. Louis, MO, USA) blocked the late phases of autophagic flux by inhibiting autophagosome fusion with lysosomes.

### 4.3. Plasmid and Transfection

The shRNA plasmid targeting AnxA6 towards the sequence of 5′-TGTGTGTGCAGCCAATGA TTTCTCGAGAAATCATTGGCTGCACACACA-3′ was constructed by Tsingke Biotechnology Co., Ltd.(Beijing, China) (shAnxA6 group). The shRNA plasmid towards the sequence of 5′-CCGGGGTTCTCCGAACGTGTCACGTCTCGAGACGTGACACGTTCGGAGAACCTTTTTGAATT-3′ served as the negative control (shCtrl group). MC3T3-E1 cells were transfected with shAnxA6 and shCtrl plasmids using Lipofectamine 8000 (Beyotime, Shanghai, China) according to the manufacturer’s protocol. After 72 h of transfection, puromycin (5 μg/mL) was used to select stably transfected shAnxA6 cells, and the final transfection efficiency was evaluated with Western blot and qRT-PCR assays.

### 4.4. FSS Loading

The FSS loading system was adapted from our previously described procedures [22,51]. Briefly, MC3T3-E1 cells were seeded onto a slide (24 × 75 mm) at a density of 5 × 10^5^ cells/mL and cultured until confluency. The cells were then immediately exposed to laminar FSS with different densities (5, 10, and 20 dyn/cm^2^) for 1 h or to 10 dyn/cm^2^ FSS for different durations (0.5, 1, 2, and 4 h) by using a parallel flow chamber that was maintained in a cell incubator with 5% CO_2_ at 37 °C. MC3T3-E1 cells without FSS treatment on slides served as the control group. For autophagic induction, MC3T3-E1 cells were preconditioned with 200 nM rapamycin for 12 h before FSS loading.

### 4.5. Transmission Electron Microscope (TEM)

The shCtrl or shAnxA6 MC3T3-E1 cells at a density of 5 × 10^5^ cells/mL were seeded on a slide (24 × 75 mm) and cultured to 90% confluence. Then, 10 dyn/cm^2^ FSS was applied to the cells for 1 h, followed by collection and centrifugation at 1500× *g* rpm for 5 min. After carefully removing the supernatant and replacing it with 0.5% glutaraldehyde for 10 min fixation at 4 °C, the samples were centrifuged for another 15 min at 13,000× *g* rpm. Cell pellets were fixed with fresh 3% glutaraldehyde and 2% OsO_4_ for 2 h in sequence, and dehydrated with graded alcohol (50%, 70%, 80%, 90%, 95%, 100%) for 15 min. Subsequently, the cells successively experienced dehydration in acetone (Amresco, Houston, TX, USA), Epon812 embedding (Amresco, Houston, TX, USA), ultrathin section (Amresco, Houston, TX, USA), and were counterstained with 3% uranyl acetate (Amresco, Houston, TX, USA) and lead citrate. Lastly, the formation of autophagosomes was observed with a transmission electron microscope (JEM1230, Jeol, Akishima, Tokyo, Japan).

### 4.6. Alizarin Red S Staining

The shCtrl or shAnxA6 MC3T3-E1 cells at a density of 5 × 10^5^ cells/mL were seeded onto a 6-well plate, cultured with osteogenic medium for 14 days, and then fixed with 95% alcohol for 15 min. Then, the cells were rinsed with ddH_2_O three times, and stained with 2% Alizarin red solution (pH 4.2, Beyotime, Shanghai, China) for 30 min at room temperature. The excess dye was removed by rinsing with ddH_2_O at least five times, and the mineralization nodules were observed with an inverted microscope (CK2, Olympus, Shinjuku, Tokyo, Japan). Each group was performed in triplicate.

### 4.7. Alkaline Phosphatase (ALP) Staining

The shCtrl or shAnxA6 MC3T3-E1 cells at a density of 5 × 10^5^ cells/mL were seeded onto a 6-well plate or a slide, cultured with osteogenic medium for 5 or 7 days, and then fixed with 4% paraformaldehyde for 15 min. After washing three times with ddH_2_O, the cells were treated with a fresh alkaline phosphatase staining work solution that had been prepared according to the manufacturer’s instructions (C3206, Beyotime, Shanghai, China) for 60 min. The staining solution was removed afterward and rinsed with ddH_2_O five times to terminate the reaction. ALP-positive cells were observed using an inverted microscope (CK2, Olympus, Shinjuku, Tokyo, Japan).

### 4.8. Alkaline Phosphatase Activity Assay

The shCtrl or shAnxA6 MC3T3-E1 cells were seeded onto slides or 6-well plates at a density of 5 × 10^5^ cells/mL, cultured with osteogenic medium for 5 days [56,57], and then lysed with a cell lysis solution (free of phosphatase inhibitors) for 30 min. The lysis solution was collected and centrifuged at 14,000× *g* rpm for 10 min, followed by determining the ALP activity of the supernatant with an Alkaline Phosphatase Assay Kit (P0321S, Beyotime, Shanghai, China) according to the manufacturer’s instructions. Then, the microplate reader was used to detect the absorbance of each group at 405 nm. Each group was performed in triplicate.

### 4.9. Western Blot Analysis

A kit was used to determine the protein concentration (P0010, Beyotime, Shanghai, China). Equal amounts of protein (20 μg) from each sample were electrophoresed on 10% or 12% sodium dodecyl sulfate-polyacrylamide gels, transferred onto polyvinylidene difluoride (PVDF) membranes and blocked with 5% skim milk for 2 h at room temperature. Then the membranes were incubated with primary antibodies (1:1000, diluted in 5% skim milk) overnight on a roller bank at 4 °C and treated with specific HRP binding secondary antibodies for 1 h at room temperature. The results were visualized via enhanced chemiluminescence and acquired with a Molecular Image^®^ ChemiDocTM XRS+system with Image Lab^TM^ 3.0 software. The ratios of the protein intensities to that of glyceraldehyde-3-phosphate dehydrogenase (GAPDH) were calculated using Image Lab 3.0 software.

### 4.10. Quantitative Real-Time PCR (qRT-PCR)

The total RNA of each sample was extracted using an RNAeasy mini Kit (RE-03111, FOREGENE, Chengdu, China) and quantified using Nanodrop 2000 (Thermo Fisher Scientific, Waltham, MA, USA). Then, 1 μg of total RNA was reversely transcribed into cDNA using the Evo M-MLV RT Mix Kit (AG11728, Accurate, Changsha, China) following the manufacturer’s instructions. SYBR^@^ Premix Ex Tag^TM^ II (TaKaRa, Kusatsu, Shiga, Japan) was used to perform qRT-PCR analysis with a Bio-rad real-time PCR system (CFX96, Bio-rad, Hercules, CA, USA). Each PCR reaction included 0.4 μM of forward and reverse primers, 100 ng of DNA, and 12.5 μL 2× SYBR Premix Ex Taq^TM^ II in a total reaction volume of 25 μL. The qPCR program included an initial denaturation step at 95 °C for 30 s, followed by 40 cycles of denaturation at 95 °C for 5 s, annealing at 60 °C for 30 s, with a final step for melting curve analysis. The mRNA expression was normalized to GAPDH and calculated using the 2^−ΔΔCt^ formula. Each experiment was performed in triplicate. The primer sequences are listed in Table 2. 

### 4.11. Immunofluorescence Staining

A total of 1 × 10^5^ cells were seeded onto a coverslip placed in 24-well plates and exposed to different FSS loadings. After different treatments, the cells were washed three times with PBS and fixed with 4% paraformaldehyde for 10 min, followed by blocking with 5% Bovine Serum Albumin (BSA) for 30 min at room temperature. Then, the samples were incubated with primary antibodies (1:500, diluted in 5% BSA) overnight at 4 °C and stained with corresponding fluorochrome-labeled secondary antibodies (1:1000, diluted in 1% BSA) for 60 min. For additional F-actin staining, FITC-phalloidin (1:200, CA1620, Solarbio, Beijing, China) was co-incubated with the cells for 30 min. The cells were subsequently stained with 4′, 6-diamidino-2-phenylindole (DAPI, 1:800) for 10 min at room temperature and rinsed five times with PBS to remove excess staining solution. The fluorescent images were captured using a Zeiss (LSM710, Oberkochen, Baden-Württemberg, GER) confocal microscope.

### 4.12. AdPlus-mCherry-GFP-LC3B Infection

AdPlus-mCherry-GFP-LC3B (C3012, Beyotime, Shanghai, China), an adenovirus expressing the mCherry-GFP-LC3B fusion protein, was used to monitor the autophagic flux in the targeted cells. The shCtrl or shAnxA6 MC3T3-E1 cells were seeded onto a coverslip placed in 24-well plates at a density of 1 × 10^5^ cells/mL and incubated with a complete DMEM medium to 50% confluence. Immediately following this, cells were treated for 24 h with a pre-configured viral solution (MOI = 20) of AdPlus-mCherry-GFP-LC3B adenovirus infection. At the end of treatment, the virus-containing medium was replaced with a fresh complete DMEM medium for another 48 h culture. After that, the transfected cells were exposed to 10 dyn/cm^2^ FSS for 1 h, followed by being rinsed three times with PBS and fixed with 4% paraformaldehyde for 15 min. Lastly, the nuclei were stained with DAPI (1:800) for 10 min and imaged with a Zeiss (LSM710, Oberkochen, Baden-Württemberg, GER) confocal microscope.

### 4.13. Statistical Analysis

Statistical analysis was performed using GraphPad Prism 9 software (GraphPad Software, San Diego, CA, USA). The data obtained in this study are presented as the mean ± standard error of the mean (SEM). Two groups were compared using the two-tailed Student’s *t*-test. Multiple groups were compared using the one-way ANOVA, followed by Tukey’s test. *p* < 0.05 was considered to be statistically significant.

## Figures and Tables

**Table 1:** Detailed information of primary antibodies.

Category	Antibody	Isotype	Manufacturer	Cat. No	Dilution
Autophagic markers	Beclin1	Rabbit pAb	Santa Cruz	sc-11427	1:100
	ATG7	Mouse mAb	Proteintech	67341-1-Ig	1:1000
	P62(SQSTM1)	Rabbit pAb	HuaBio	R1309-8	1:1000
	ATG5	Rabbit mAb	HuaBio	ET1611-38	1:1000
	LC3B	Rabbit mAb	Abcam	Ab192890	1:1000
Osteoblastic differentiation marker	ALP	Rabbit pAb	HuaBio	ET1601-21	1:500
	Col I	Rabbit pAb	Proteintech	14695-1-AP	1:1000
	Runx2	Rabbit mAb	CST	#12556	1:1000
Annexin	AnxA6	Rabbit pAb	Proteintech	12542-1-AP	1:500 for IF 1:1000 for WB
	GAPDH	Rabbit mAb	SAB	21337	1:1000

**Table 2:** Primer sequences for qRT-PCR detection.

Name	Forward	Reverse
GAPDH	5′-AACATCAAATGGGGTGAGGCC-3′	5′-GTTGTCATGGATGACCTGGC-3′
AnxA6	5′-CTTCGGCAGTGACAAGGAGT-3′	5′-TGGCGTCACAATAGGCAAGT-3′

## Data Availability

The study did not report any data.
