# Correction: Pei et al. Fluid Shear Stress Regulates Osteogenic Differentiation via AnnexinA6-Mediated Autophagy in MC3T3-E1 Cells. *Int. J. Mol. Sci.* 2022, *23*, 15702

**DOI:** 10.3390/ijms27104287

**Published:** 2026-05-12

**Authors:** Tong Pei, Guanyue Su, Jie Yang, Wenbo Gao, Xinrui Yang, Yaojia Zhang, Jie Ren, Yang Shen, Xiaoheng Liu

**Affiliations:** Institute of Biomedical Engineering, West China School of Basic Medical Sciences & Forensic Medicine, Sichuan University, Chengdu 610041, China

## Figure Correction

In the original publication [1], there was a mistake in Figure 3D “Representative images show alizarin red staining after 14 days of osteogenic induction. Calcified nodules were shown as red staining.” as published. The image of Alizarin red staining representing the treated group (shAnxA6) was inadvertently replaced with an image from a different experimental group. In detail, the PH.D. students participating in Annexin Project are simultaneously working on two research topics, one is Annexin A5 and the other is Annexin A6. Due to the concurrent nature of these projects, a shAnxA5 image was mistakenly selected and placed into the shAnxA6 panel during figure assembly. The revised Figure 3 appears below.

The authors state that the scientific conclusions are unaffected. This correction was approved by the Academic Editor. The original publication has also been updated.

## Figures and Tables

**Figure 3:**
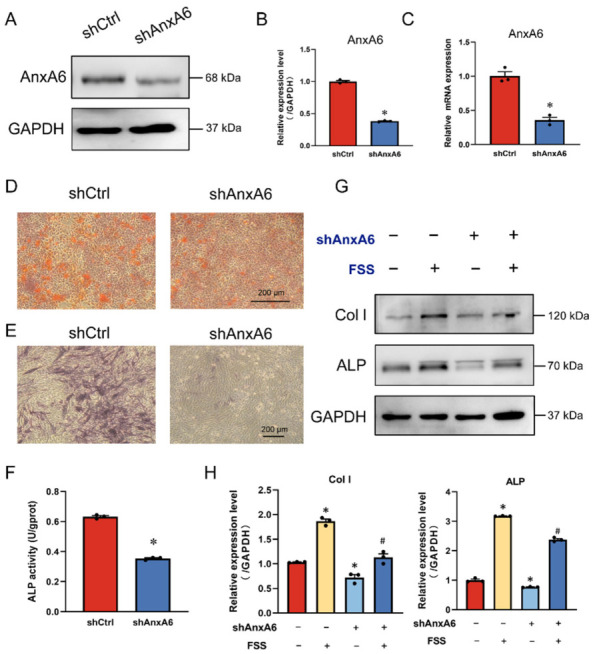
AnxA6 is involved in FSS-induced osteogenic differentiation. (**A**,**B**) Western blot analysis and quantification of AnxA6 expression in MC3T3-E1 cells transfected with negative shRNA (shCtrl) or AnxA6 shRNA (shAnxA6). GAPDH served as an internal control (*n* = 3). (**C**) The gene expression of AnxA6 was detected with qRT-PCR and quantified with GAPDH normalization (*n* = 3). (**D**) Representative images show alizarin red staining after 14 days of osteogenic induction. Calcified nodules were shown as red staining. (**E**) Representative images of ALP staining after 5 days of osteogenic induction. ALP-positive cells shown as blue staining. (**F**) Quantification of ALP activity in shCtrl and shAnxA6 MC3T3-E1 cells with 1 h of 10 dyn/cm^2^ FSS loading (*n* = 3). (**G**,**H**) Western blot analysis and quantification of osteogenic protein expression in shCtrl and shAnxA6 MC3T3-E1 cells with or without 1 h of 10 dyn/cm^2^ FSS loading (*n* = 3). GAPDH served as an internal control (*n* = 3). All data are presented as mean ± SEM. * *p* < 0.05 versus static shCtrl group; # *p* < 0.05 versus FSS group.
